# Acute respiratory distress in Maniitsoq, Greenland

**DOI:** 10.1186/1757-7241-20-S2-O1

**Published:** 2012-04-16

**Authors:** Patricia Duch, Niels E Ebbehøj, Folmer Lynggaard

**Affiliations:** 1 Dept. of Internal Medicine Dronning Ingrids Hospital, Nuuk, Greenland; 2 Dept. of Occupational Medicine, Bispebjerg University Hospital, Denmark

## Background

Inhalation of aerosols of surface active substances is a known risk factor for development of acute respiratory distress syndrome (ARDS). A number of small case series are reported with exposure for aerosols from spray-cans with textile or leather protecting agents. We report a series of cases with respiratory symptoms in a group of 39 persons exposed to a floor-sealing product, Anti Fleck Super(R) (AFS).

Case: In 2010, a supermarket in the Greenlandic town Maniitsoq, was under renovation.

A 300 m2 floor was sprayed with AFS. The spraying created a fog of aerosols of poly-fluorinated silicones. Craftsmen, employees, and customers inhaled the aerosols during a period of minutes to 3 hours. Within hours 39 persons developed cough, dyspnoea and fever in varying degrees.

It was necessary to evacuate the 39 patients with progressive symptoms, by airplane from the small hospital in Maniitsoq, to the well-equipped Dronning Ingrids Hospital (DIH), Nuuk. Triage was used.

## Methods

Symptoms were recorded in Maniitsoq and DIH. All patients had arterial blood analyses and chest x-rays taken on arrival to DIH. Two months after the incident, the patients were offered a thoroughly clinical follow-up with lung function test, oxygen-saturation working test and chest x-ray.

The patients: male and female, mean age 33 years, mainly without earlier medical record. Almost all were smokers.

## Results

Within few hours 39 persons developed symptoms of respiratory distress.

Mainly difficulty breathing, coughing, flu-like symptoms, tachycardia, fever and reduced oxygen saturation level. Seven patients had acute lesions on chest X-ray.

All symptoms peaked and virtually subsided within 24 hours. Except in 3 patients, hospitalized in intensive care, who had severe respiratory symptoms for 48 hours, Sat. average 80%. At clinical follow-up, 15 patients still experienced difficulty breathing during hard physical work. But all had normal medical examinations including normal saturation during exercise.

## Conclusion

The Greenlandic healthcare system has a set-up that makes it capable of managing acute situations even up to a larger scale. Evacuation and the management in the hospitals functioned optimally throughout the acute situation.

Poly-fluorinated silicones are potent chemicals able to produce pulmonary toxicity even at low-intensive exposure levels.

